# Robotic navigational bronchoscopy in a thoracic surgery practice: Leveraging technology in the management of pulmonary nodules

**DOI:** 10.1016/j.xjon.2023.07.004

**Published:** 2023-07-17

**Authors:** Andrew R. Brownlee, Justin J.J. Watson, Akbarshakh Akhmerov, Shruthi Nammalwar, Qiudong Chen, Sevannah G. Soukiasian, Harmik J. Soukiasian

**Affiliations:** Division of Thoracic Surgery, Department of Surgery, Cedars Sinai Medical Center, Los Angeles, Calif

**Keywords:** robotic navigational bronchoscopy, robotic thoracic surgery, pulmonary nodules

## Abstract

**Objectives:**

Robotic navigational bronchoscopy is increasingly used to improve diagnostic yield for pulmonary nodules compared with the 50% to 60% obtained by standard bronchoscopy; however, safety and efficacy data are limited to small series. The aim of this study was to evaluate diagnostic yield and clinical outcomes in a large multisurgeon single-center cohort.

**Methods:**

All patients who underwent robotic navigational bronchoscopy and biopsy from September 2020 to October 2022 were identified from a prospective institutional registry. The primary outcome was diagnostic yield. The secondary outcome was diagnostic yield for molecular testing.

**Results:**

A total of 503 nodules were biopsied during the study period. Median nodule size was 2.1 cm. Overall diagnostic yield was 87.9%. Factors associated with increased diagnostic yield were decreased time from date of planning computed tomography to procedure date (odds ratio, 0.98; 95% CI, 0.96-0.99; *P* = .04) and greater nodule size (odds ratio, 1.03; 95% CI, 1.01-1.07; *P* = .02) per 0.1-cm increment. Molecular analysis was sent in 101 patients and was sufficient in 90% of cases. Complications occurred in 22 (5%) patients, including 13 (3.1%) with pneumothoraxes (7 patients requiring a chest drain), and 5 (1.2%) patients had bleeding requiring intraprocedural bronchial intervention. A total of 41 patients were consented for biopsy and resection during a single anesthetic event. Four of these cases were stopped at robotic navigational bronchoscopy due to an alternative diagnosis. Mean length of stay was 3.4 ± 1.1 days. There were no major complications.

**Conclusions:**

This study suggests robotic navigational bronchoscopy has a high diagnostic yield and obtains adequate tissue for molecular analysis critical for selection of targeted therapies. With careful patient selection robotic navigational bronchoscopy can be combined with surgery to treat lung cancer as a single procedure with low complication rates.

**Figure:**
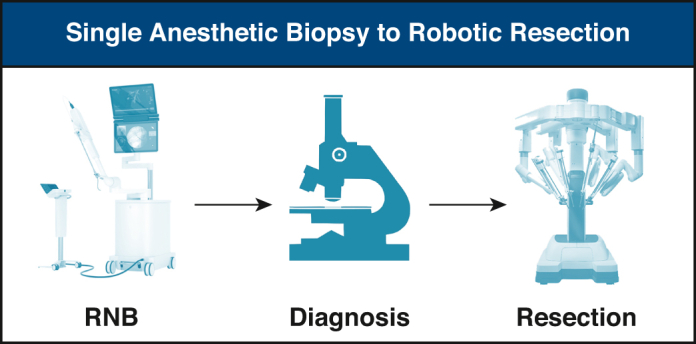
Robotic navigational bronchoscopy combined with surgical resection during the same anesthetic event.

**Central Message**

We demonstrate our experience with robotic navigational bronchoscopy and its integration into a busy thoracic surgical oncology practice, while combining it with surgical resection when appropriate.

**Perspective**

In this study, we show that robotic navigational bronchoscopy can be leveraged as an integral component of a busy thoracic surgical oncology program. The high diagnostic yield even for small peripheral nodules as well as its low complication rate allows it to be effectively and efficiently employed in combination with surgical resection while maintaining good oncologic principles.

See Discussion on page 7.

Lung cancer is the most common cause of cancer-related death in both the United States and worldwide.^1^ Overall 5-year survival is 22%, whereas survival of early-stage lung cancer can be up to 90%. Greater than 70% of lung cancers are locally advanced or metastatic at the time of diagnosis, resulting in the observed decreased overall survival.^2^ Lung cancer screening can diagnose nodules in their earlier stages, thus improving survival. However, only 5.8% of eligible individuals are currently screened in the United States.^3^, ^4^, ^5^

With widespread adoption of lung cancer screening, and increased incidental detection, the total number of nodules requiring surveillance and sampling will increase.^6^ Most of these nodules (>70%) are in the outer one-third of the lung^7^ and are subcentimeter (50.8% of non–small cell lung cancer diagnosed in the National Lung Screening Trial).^4^ These smaller, more peripheral nodules are more difficult to diagnose, posing a challenge for clinicians who must balance the benefit of a potential diagnosis against the risk of invasive diagnostic procedures with potential complications.

Robotic navigational bronchoscopy (RNB) is a minimally invasive endoluminal platform used in the diagnosis of lung nodules. There are 2 main types of commercially available RNB: one utilizes shape sensing robotic bronchoscopy (ssRNB) and the other uses electromagnetic navigational bronchoscopy. The adoption of RNB has increased significantly in part due to its superiority over standard transbronchial biopsy and other navigational technologies, with yields of 44% to 74%.^8^, ^9^, ^10^, ^11^, ^12^ The Clinical Evaluation of superDimension Navigation System for Electromagnetic Navigation Bronchoscopy trial using electromagnetic RNB improved the diagnostic rate to 67.8%,^13^^,^^14^ whereas a more recent series using ssRNB reported a diagnostic yield of 82%.^15^ However, safety and efficacy data are limited to small series.

In this study, we provide the largest single-center experience in the use of ssRNB. The aim of this study was to evaluate diagnostic yield and clinical outcomes in a large single-center, multisurgeon experience. Additionally, we report our rate of adequate tissue acquisition for molecular analysis. A select subset of patients underwent a combined procedure with single anesthesia bronchoscopy to robotic resection (SABRR) of the lesion. Our early experience and outcomes combining these approaches are also reported.

## Methods

### Study Design

Patients were included from both thoracic surgery and interventional pulmonology services. All patients referred to Cedars-Sinai Medical Center for evaluation of pulmonary nodules were individually assessed by the practitioner for the need for biopsy and candidacy for RNB. All RNB procedures performed between September 2020 and October 2022 were captured in our prospective database and analyzed retrospectively. RNB procedures performed for diagnostic sampling of 1 or more pulmonary lesions were included in the final analysis. Patients who were consented for concomitant resection in the same anesthesia event (eg, SABRR) were also included. This study was approved by Cedars-Sinai institutional review board (STUDY00001670; approved September 15, 2021). The need for informed consent was waived.

### Target Sampling and Lesion Characterization

Pulmonary parenchymal lesions were defined as being bordered by lung parenchyma requiring transbronchial sampling. The widest lesion diameter and consistency were documented based on the planning computed tomography (CT) imaging in the axial, coronal, and sagittal planes. Lesion location was defined as the inner one-third, the middle one-third, and outer one-third of the lung parenchyma, respectively, as previously described.^15^ An airway leading into or through the lesion was designated as a positive bronchus sign. Thirty-one procedures in patients with no discrete nodules and a diffuse parenchymal consolidative process who underwent RNB were excluded from median lesion size analysis because no discrete nodule size could be determined.

RNB was performed using the Ion Robotic-Assisted Endoluminal Platform (Intuitive Surgical). CT scan planning was conducted using PlanPoint software (Intuitive Surgical). All procedures were performed in a dedicated endoscopy suite or operating room by a thoracic surgeon or interventional pulmonologist. Procedure time was documented from procedure start to procedure stop time. A flexible bronchoscope was used to first inspect the airways and position the endotracheal tube. The RNB catheter and vision probe were then inserted into the endotracheal tube and registration was performed synchronizing the real-time and virtual bronchoscopy views. Once the catheter was navigated to the target lesion the vision probe was removed and proximity was confirmed using 2-dimensional fluoroscopy and radial endobronchial ultrasound probe (rEBUS) as needed. Successful navigation was defined as achieving catheter-target proximity that allowed lesion sampling. Sampling was performed using a 19-, 21-, or 23-guage Flexision needle (Intuitive Surgical). Pathology evaluation was performed in the central lab for all procedures utilizing runners because no rapid onsite evaluation was available.

For patients who were scheduled for SABRR, the first steps of the RNB proceeded as above. The next steps were dependent on pathology results of the RNB. The cytology results and frozen section results were reviewed in detail with the pathologist and based on the findings, a consensus was reached between the surgeon and pathologist on whether to proceed with resection, or to conclude the case and wait for final pathology. If consensus was made to proceed with resection, the robotic instruments were opened. An EBUS with lymph node biopsy was performed if indicated. The single lumen endotracheal tube was exchanged for a double lumen tube and an arterial line was placed. The patient was placed in the lateral position and a robotic resection was performed.

### Outcomes

The primary outcome of this study was diagnostic yield of RNB sampling per individual lesion. We defined 4 categories of diagnosis malignant; nonmalignant, including inflammatory or infectious etiologies; atypical cells suspicious for a malignant or premalignant lesion; and insufficient sampling. A benign diagnosis was given if the lesion demonstrated resolution on repeat imaging, was confirmed with repeat tissue sampling or had 1 year of radiographic stability. Our secondary end point was diagnostic yield for molecular testing. Safety was evaluated with 30-day postoperative follow-up. All patients who had a nondiagnostic procedure were evaluated for appropriate follow-up (Table 1). We also evaluated the utility and safety when combined with concomitant surgical resection during the same anesthetic event, including rate subsequent surgery was stopped due to an alternative diagnosis on RNB. In select patients who proceeded with SABRR, 30-day outcomes were evaluated, including postoperative mortality, complications and length of stay (Table 2).

**Table 1:** Results of nondiagnostic procedures (N = 41)

Outcomes following a non-diagnostic procedure	Result
Imaging surveillance ongoing	10
Computed tomography-guided biopsy with diagnosis	3
Repeat Ion with diagnosis	3
Future surgery	12
Concomitant surgery	5
Benign based on lesion, resolution, or completion of surveillance	7
Lost to follow up	0

*Ion*, Ion Robotic-Assisted Endoluminal Platform (Intuitive Surgical).

**Table 2:** Patients who went on to surgery (n = 110)

Surgical procedure	Result
Mediastinoscopy	2 (1)
Pleural Biopsy	2 (1)
Wedge Resection	22 (20)
Segmentectomy	17 (15.4)
Lobectomy	67 (60.9)

Values are presented as n (%).

### Statistical Analysis

Descriptive statistics were presented as counts and percentages for categorical variables and as medians and interquartile range for continuous variables. All statistical tests were 2-tailed. SPSS version 24.0 (IBM-SPSS Inc) and SAS version 9.4 (SAS Institute Inc) was used for analyses. To identify factors associated with diagnostic yield, we performed univariate logistic regression analysis on a nodule-level dataset using generalized estimating equation modeling with a logit link function and binomial distribution, accounting for patient-level clustering.

## Results

A total of 503 nodules were biopsied during 415 procedures in 407 patients during the study period. Forty-nine percent of cases were performed by thoracic surgery and the remainder by interventional pulmonary. Forty-one patients were consented for concomitant SABRR with 4 stopped due to an alternative diagnosis not requiring surgical intervention. Patient demographics and clinical characteristics are summarized in Table 3. The mean age was 69.3 ± 12.3 years and 50.3% were women. Most patients were ever smokers 62.8%. Median nodule size was 2.1 cm, 50.3% were in the upper lobes, and 51.3% of lesions were located in the peripheral on-third of the lung. A bronchus sign was present in 24.4%. Nodules were solid in 59.2%, mixed solid 11.1%, and pure ground glass in 11.7%. The mean procedure time was 67 ± 30 minutes. Successful navigation to the target lesion was achieved in 99%. Four patients were not able to undergo successful navigation to the lesion: 1 for prior pneumonectomy and inability to complete registration and 3 others due to anatomy precluding successful navigation. Intraoperative imaging included radial EBUS in 57.4% and 2-dimensional fluoroscopy in 100%. Transbronchial forceps biopsy was used in 94% and linear EBUS was performed in 38.6%.

**Table 3:** Robotic navigational bronchoscopy data

Variable	Result
Clinical characteristics (n = 415)	
Age (y)	69.3 ± 415
Female gender	50.3 (209)
Smoking status	
Current	8.6 (36)
Former	54.2 (225)
Never	37.1 (154)
Lesion/procedure characteristics (n = 503)	
Median lesion size (cm)	2.1
Lesions distribution	
RUL	32.0 (161)
LUL	18.3 (92)
RML	10.3 (52)
RLL	26.0 (131)
LLL	13.3 (67)
Parenchymal location	
Peripheral	51.3 (258)
Middle	33.4 (168)
Central	15.3 (77)
Bronchus sign (n = 495)	
Yes	24 (121)
No	76 (374)
No. of lesions targeted per procedure	1.26 ± 0.5
Procedure time (min)	67 ± 30
Radial probe EBUS (%) (n = 502)	57.4 (288)
Nodule type	
Solid	59.2 (298)
Part solid	11.1 (56)
Cavitary	6.5 (33)
Consolidation	11.1 (56)
GGO	11.7 (59)
Biopsy results	
Diagnostic yield (%)	87.9
NGS yield (%)	90
Diagnostics	
Primary lung cancer	42.5 (188)
Metastasis	7.4 (33)
Other malignant	2.4 (11)
Other benign	0.9 (4)
Premalignant	2.9 (13)
Inflammatory	12.4 (55)
Infectious	31.2 (138)
Nondiagnostic	12.1 (61)
Complication	
Overall rate	5.3 (22)
Pneumothorax rate	3.1 (13)
Chest tube placed	1.7 (7)
Bleeding	1.2 (5)
Infection	0.7 (3)
CVA	0.2 (1)

Values are presented as mean ± SD or % (n) unless otherwise noted. *RUL*, right upper lobe; *LUL*, left upper lobe; *RML*, right middle lobe; *RLL*, right lower lobe; *LLL*, left lower lobe; *EBUS*, endobronchial ultrasound; *GGO*, ground glass opacity; *NGS*, next generation sequencing; *CVA*, cerebrovascular accident.

Overall diagnostic yield was 87.9%. The diagnostic yield for nodules ≤1 cm was 83%, 1.1 to 2 cm, 87% and > 2 cm it was 93% (*P* = .11). The positive and negative predictive values were 100% and 91.6%, respectively, with a 6.8% false negative rate. On univariate analysis, factors associated with increased diagnostic yield were decreased time per day from date of planning CT to procedure date (odds ratio, 0.98; 95% CI, 0.96-0.99; *P* = .04) and greater nodule size (odds ratio, 1.03; 95% CI, 1.01-1.07; *P* = .02) per 0.1-cm increment (Table 4). Molecular analysis was sent in 101 patients and was sufficient in 90% of cases. There were no mortalities. Complications occurred in 22 (5%) patients, including 13 (3.1%) with pneumothoraxes (7 patients requiring a chest drain), and 5 (1.2%) patients had bleeding requiring a bronchial intervention. Three patients developed postoperative pneumonia and one patient had a cerebrovascular accident. Subsequent anatomic pulmonary resection was performed in 84 (21%) patients. Benign resections were performed in 9 patients (8.5%: 4 wedges resections, 5 segmentectomies, and 0 lobectomies).

**Table 4:** Univariate logistic regression

Variable	Odds ratio	95% CI	*P* value
Mean nodule size, per 1-mm increase	1.37	1.05-1.78	.02
Time since Ion∗ CT, per 1-d increase	0.98	0.96-0.99	.04
Air bronchus sign	1.44	0.7-2.97	.32
Radial EBUS use	0.75	0.4-1.42	.38
Prior anatomical lung resection	1.17	0.34-4.04	.8
Nodule location			
Central	Ref	Ref	
Middle	0.5	0.19-1.35	.17
Peripheral	0.72	0.27-1.94	.52
Nodule type			
Solid or semisolid	Ref	Ref	
Mixed	0.86	0.35-2.07	.73
Infiltrate	1.42	0.31-6.44	.65
GGO	0.99	0.40-2.46	.99
Consolidation	0.85	0.27-2.64	.78
Cavitary	0.93	0.31-2.74	.89
Nodule lobe			
RUL	Ref	Ref	
RML	1.4	0.51-3.80	.52
RLL	1.73	0.84-3.54	.13
LUL	2.1	0.92-4.82	.08
LLL	0.88	0.41-1.89	.75

*CI*, Confidence interval; *CT*, computed tomography; *EBUS*, endobronchial ultrasound; *GGO*, ground glass opacity; *RUL*, right upper lobe; *RML*, right middle lobe; *RLL*, right lower lobe; *LUL*, left upper lobe; *LLL*, left lower lobe; *Ref*, reference category.

∗ Ion Robotic-Assisted Endoluminal Platform (Intuitive Surgical).

A total of 61 patients had nondiagnostic initial RNB: 10 of whom are undergoing active surveillance, 3 underwent repeat RNB with diagnosis, 3 underwent subsequent CT-guided biopsy with diagnosis, and 12 underwent a subsequent surgery. Five of the SABRR cases were nondiagnostic on RNB, and diagnosis was obtained surgically in the same setting. Seven were determined benign due to lesion resolution or completion of recommended surveillance. Twenty patients had a benign diagnosis, but have not completed 1 year of surveillance. No patients were lost to follow-up.

SABRR was considered in 41patients (Table 5). The diagnostic yield for these cases was 85%. Four of the SABRR cases were stopped due to an alternative diagnosis not requiring surgical intervention: lymphoma, metastatic adenocarcinoma, pneumonia and granuloma. These findings were confirmed on final pathology. A total of 37 patients went on to have a SABRR procedure. The mean operative time for these cases was 253 ± 59 minutes. The benign resection rate in the series improved with our learning curve, with the overall benign resection rate being 16%. During our experience validating this technology in a clinical setting our initial benign resection rate was 25% and then decreased to 5% in our last 20 cases. There were no mortalities in this subgroup. Mean chest tube duration was 2.4 ± 0.7 days and postoperative length of stay was 3.4 ± 1.1 days. There were 3 (8.1%) patients with prolonged air leak. There were no major complications and no patients were taken back to the operating room. There was 1 readmission for hydropneumothorax resolved with a chest drain.

**Table 5:** Single anesthesia biopsy to resection

Clinical characteristics (N = 41)	Result
Pulmonary function tests	
FEV1	101.8%
DLCO	82.9%
Comorbidities	
CAD	17.1 (7)
MI	2.4 (1)
HTN	61 (25)
CHF	0 (0)
PVD	2.4 (1)
CVA	4.9 (2)
DM	21 (9)
CKD	4.9 (2)
Procedure characteristics	
Total No. of planned procedures	41
Benign resection rate/series (%)	16
Benign resection rate/last 20 patients (%)	5
Mean operative time (min)	253 ± 59
Mean chest tube duration (d)	2.4
Mean length of stay (d)	3.4
Complications: Prolonged air leak (>5 d)	3
30-d readmissions	2.4 (1)
Mortality	0 (0)

Values are presented as mean ± SD or % (n) unless otherwise noted. *FEVI*, Forced expiratory volume in 1 second; *DLCO*, diffusing capacity of lungs for carbon monoxide; *CAD*, coronary artery disease; *MI*, myocardial infarction; *HTN*, hypertension; *CHF*, congestive heart failure; *PVD*, peripheral vascular disease; *CVA*, cerebrovascular accident; *DM*, diabetes mellitus; *CKD*, chronic kidney disease.

## Discussion

The number of pulmonary nodules discovered incidentally and via screening will continue to increase. RNB historically has had varied adoption rates and efficacy. As the technology continues to improve, physicians will be able to leverage this platform to more confidently diagnose previously difficult to access pulmonary nodules, such as smaller, more peripheral nodules. Lesions with a high pretest probability of malignancy may be considered for a SABRR.

This is the largest reported experience using the ssRNB platform. The feasibility, diagnostic yield and safety using ssRNB in our study compared favorably to other reported experiences. Furthermore, it was safe with minimal complications and no 30-day mortality. Diagnostic yield did not significantly decrease with subcentimeter nodules, with a diagnostic rate of 83% for lesions ≤1 cm allowing diagnosis at an earlier stage. Peripheral location of nodules did not influence diagnostic yield. Adequacy of specimens for molecular analysis was 90% in cases where tissue sampling was requested. This is also an extremely important aspect to any technology used in the diagnosis of non–small cell lung cancer because more patients will be receiving immunotherapy, either in the adjuvant, neoadjuvant, or definitive setting.

This is the first reported study to show that ssRNB technology can be seamlessly integrated into a busy thoracic surgical oncology practice, leveraging RNB and anatomic lung resection in a single anesthetic event. We demonstrate that SABRR can effectively be used for the diagnosis, staging, and definitive management of pulmonary nodules. This technique can be done safely in a multistep procedure without lengthy anesthetic times. The ability to perform same-day diagnosis and treatment has the potential to reduce wait times in appropriately selected patients.

Our benign resection rate in the SABRR group decreased with time. The explanation for this is likely multifactorial, but attributed in part to increased confidence in RNB diagnostic results for both benign and malignant lesions. In our initial experience, a benign RNB diagnosis often led us to confirm this finding with surgical resection. In these cases, we had 100% concordance between RNB pathology and surgical pathology. This in turn gave us the internal validity to stop after RNB if the biopsy result precluded proceeding with surgical resection even in a cohort of patients with a high pretest probability of malignancy.

This study has some limitations. This was a retrospective observational study at a single institution. Selection bias for patients undergoing RNB may be present. Our study includes a multidisciplinary team of interventional pulmonologists and thoracic surgeons and disparity in yield and outcomes between specialties and proceduralists was not evaluated. Finally, the diagnosis of benign lung lesions will require ongoing long-term follow-up to definitively determine accuracy.

## Conclusions

We demonstrate our initial experience with the ssRNB platform in our first 503 nodules with excellent diagnostic yield, safety, and feasibility data even with subcentimeter nodules in the periphery of the lung. We also demonstrate the feasibility and safety of SABRR in a high-volume thoracic oncology surgical program.

### Webcast

You can watch a Webcast of this AATS meeting presentation by going to: https://www.aats.org/resources/value-of-robotic-navigational-bronchoscopy-to-enhance-diagnostic-yield-and-guide-oncological-strategy-in-treatment-of-pulmonary-nodules.

**Figure:**
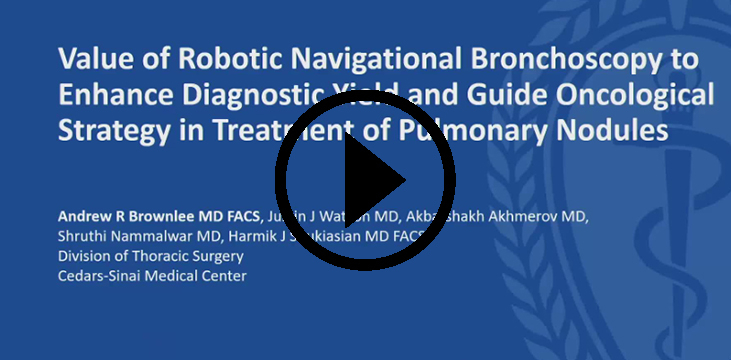

### Conflict of Interest Statement

Drs Soukiasian and Brownlee are proctors for Intuitive. All other authors reported no conflicts of interest.

The *Journal* policy requires editors and reviewers to disclose conflict of interest and to decline handling or reviewing manuscripts for which they have a conflict of interest. The editors and reviewers of this article have no conflicts of interest.
